# The Pressure on the Profession

**Published:** 1916-03-11

**Authors:** 


### The Hospital

The Workers' Newspaper of Administrative Medicine and Institutional Life, Administration, National Insurance and Health.

No. 1552, Vol. LIX. SATURDAY, MARCH 11, 1916. PRICE ONE PENNY.

### THE PRESSURE ON THE PROFESSION.

The rapid calling to the Colours of men who have attested under Lord Derby's scheme means an increasing demand for more doctors by the medical authorities of the Army; and the pressure exercised on the profession is consequently an increasing quantity. Neither men in the camps nor men in the trenches can be left without the succour of medical aid, and no one is more ready to admit this than the doctors themselves. The present war has emphasised and heightened the part played by the medical profession in relation both to the organisation of the Army and to its activities in the field, and this part in the future will grow greater rather than less. It is therefore not surprising that the War Office says "We must have all the doctors which we need." No one contests this proposition, but in not a few quarters it is questioned whether the demands of the authorities are not in excess of their needs, and the volunteers would be more numerous were the conviction general that those already serving are wisely utilised. The Central War Committee stands, as one may say, between those who ask and those who hesitate; and its aim is to overcome the hesitations and to supply the Director-General with the help he says he requires. Unhappily this Committee came into existence in circumstances which give an opportunity for adverse comment. It was not elected with any mandate from the general body of the profession, but was created at a private meeting of a particular organisation. Hence its authority is under challenge. Further, there is a suspicion that in its action it has shown itself unduly complaisant to the powers that be, and has accepted the official demands without scrutiny or investigation. Now we hold no brief for the Committee or for the organisation which promoted its appointment. Nor do we for a moment profess to regard all its actions with admiration or approval. Still less do we say that a better organisation and more efficient machinery could not have been obtained. But it is necessary for everyone to remember the urgencies of the situation. Were we dwelling in the leisurely days of peace many things might be refused or denied which to-day patriotism calls upon us to accept. And the Central War Committee, it seems to us, is among the number. Allow that its birth was not a happy one and that its composition is not ideal. Allow, also, that supreme wisdom has not marked all its operations. Still it exists, and it exists as the sole piece of machinery by which the profession in its corporate capacity can speak to the Army and can secure, at least in some degree, that the reasonable conditions of the profession shall receive attention. It may be said that the Universities, or the Royal Colleges, or the medical schools are more suitable avenues through which the profession could express its wishes. Even if this is correct, the broad fact stands that after twelve months of war none of these organisations had in England betrayed any manifest appreciation of the responsibility now thrust on them by their admirers. If they possessed the necessary qualities these existed in a latent form, and even the excursions and alarms of war had failed to arouse them into activity. In these circumstances it is surely fortunate that someone moved, and so far at least there is cause for gratitude. If other organisations remained in an appropriate slumber it would seem wise in the circumstances to make the best of the one which showed evidence of being awake, even if choice in the matter is on the limited scale associated with the name of Tobias Hobson. Certainly there can now be no question of a rival or alternative committee, and were the existing one to disappear the War Office, having no professional organisation with which to negotiate, would be driven to deal with practitioners in their individual capacities. And the scorpions of the War Office would hardly be more welcome than the whips of the Central War Committee.

The most recent development of military policy has made conspicuous the need for some professional organisation to represent the position and interests of medical practitioners. Under the

Military Service Act unmarried doctors of military age come, like other men of the same age and status, within the purview of the local tribunals, and any claim for exemption must be presented to one or other of these tribunals. In at least one instance this position has been demonstrated by experience, and it may be doubted whether the applicant would care to repeat the trial. Through the agency of the Central War Committee such a necessity can be avoided, for the War Office will accept the Committee's decision. At least this is the position at present; but should the supply of doctors fail, some other form of pressure will certainly be put into operation. The Committee stands as an apparatus which secures professional judgment for professional questions, and whatever be its demerits, it is to be preferred to the rough verdict of a lay tribunal.

There is at the basis of the whole transaction the question of confidence in the Committee; and the Committee may reasonably be called on to show cause in this direction. Especially, at least so it seems to us, is there room for more publicity in its proceedings. It has to convince the doubters that men whose names represent other interests than a particular Association really attend the meetings; that the Army authorities are pressed to utilise fully and wisely the doctors who are already serving; and that the needs of the civil population are receiving due protection. When these ends are attained, questions related to the birth of the Committee will sink into insignificance.

